# Treatment of Diabetes Mellitus by Balsam of Peru

**Published:** 1846-10

**Authors:** 


*Treatment of Diabetes Mellitus by Balsam of Peru.*—Dr. Vauhes reports in the *Journ. des Connaiss. Med. Chirurg.*, a case of diabetes mellitus cured in five weeks by balsam of Peru. The medicine was given in the dose fifty drops four times a day, and increased to five drachms daily.—*Medical News.*

